# Integrated Metabolome and Transcriptome during Fruit Development Reveal Metabolic Differences and Molecular Basis between *Lycium barbarum* and *Lycium ruthenicum*

**DOI:** 10.3390/metabo13060680

**Published:** 2023-05-23

**Authors:** Ziyang Xie, Yu Luo, Changjian Zhang, Wei An, Jun Zhou, Cheng Jin, Yuanyuan Zhang, Jianhua Zhao

**Affiliations:** 1 Sanya Nanfan Research Institute of Hainan University, Hainan Yazhou Bay Seed Laboratory, Sanya 572025, China; 2 College of Tropical Crops, Hainan University, Haikou 570228, China; 3 National Wolfberry Engineering Research Center, Wolfberry Science Research Institute, Ningxia Academy of Agriculture and Forestry Sciences, Yinchuan 750002, China; 4 College of Biological Science and Engineering, North Minzu University, Yinchuan 750021, China

**Keywords:** *Lycium barbarum*, *Lycium ruthenicum*, metabolome, transcriptome, flavonoids

## Abstract

Wolfberry (*Lycium barbarum*) is a traditional cash crop in China and is well-known worldwide for its outstanding nutritional and medicinal value. *Lycium ruthenicum* is a close relative of *Lycium barbarum* but differs significantly in size, color, flavor and nutritional composition. To date, the metabolic differences between the fruits of the two wolfberry varieties and the genetic basis behind them are unclear. Here, we compared metabolome and transcriptome data of two kinds of wolfberry fruits at five stages of development. Metabolome results show that amino acids, vitamins and flavonoids had the same accumulation pattern in various developmental stages of fruit but that *Lycium ruthenicum* accumulated more metabolites than *Lycium barbarum* during the same developmental stage, including L-glutamate, L-proline, L-serine, abscisic acid (ABA), sucrose, thiamine, naringenin and quercetin. Based on the metabolite and gene networks, many key genes that may be involved in the flavonoid synthesis pathway in wolfberry were identified, including *PAL*, *C4H*, *4CL*, *CHS*, *CHI*, *F3H*, *F3’H* and *FLS*. The expression of these genes was significantly higher in *Lycium ruthenicum* than in *Lycium barbarum*, indicating that the difference in the expression of these genes was the main reason for the variation in flavonoid accumulation between *Lycium barbarum* and *Lycium ruthenicum*. Taken together, our results reveal the genetic basis of the difference in metabolomics between *Lycium barbarum* and *Lycium ruthenicum* and provide new insights into the flavonoid synthesis of wolfberry.

## 1. Introduction

*Lycium barbarum* (also known as red wolfberry) is an important cash crop in northwest China and is popular with consumers because of its rich nutritional and medicinal value [1]. *Lycium barbarum* belongs to the genus *Lycium* in the *Solanaceae* family, which contains 97 species and is an important medicinal and food plant [2]. Many medical studies have shown several pharmacological benefits of wolfberry fruits, including skin hydration, blood sugar regulation, cancer prevention, antiaging effects and immune system enhancement [3]. Research on wolfberry is beneficial to the cultivation of varieties with high nutritional quality, which can increase economic value.

Metabolites are considered to be the bridge between genotype and phenotype, and changes in metabolite levels can directly reveal the function of genes, thus revealing biochemical and molecular mechanisms more effectively [4]. A series of metabolites have been identified in various organs of *Lycium barbarum* [5]. For example, rutin, quercetin and kaempferol are the main flavonoid metabolites in *Lycium barbarum*. Recently, 13 kinds of flavonoid compounds were identified from the fruits of *Lycium barbarum*, among which daphnetin, 6,7-dihydroxycoumarin, astragalin, taxifolin, eriodictyol, naringenin and chrysoeriol were identified for the first time in the fruits of *Lycium barbarum*, which greatly enriched the kinds of flavonoids in the fruits of *Lycium barbarum* [6]. Wolfberry fruits are also rich in vitamins, providing twice the recommended daily dose of vitamins A and C [7]. Wolfberry fruits are especially rich in *Lycium barbarum* polysaccharide, an antioxidant. *Lycium barbarum* polysaccharide is a key bioactive element in fruits and is beneficial to human health [8,9]. *Lycium barbarum* polysaccharides are reported to have antitumor activity (anticancer) and can effectively prevent diabetes and hyperglycemia [10,11,12]. The main fatty acids of wolfberry fruits are linoleic acid, followed by oleic acid, palmitic acid and stearic acid (about 95% of the total fatty acids) [13,14,15,16]. Wolfberry fruits also contain a lot of free amino acids, including L-aspartic acid (Asp), glutamic acid (Glu), glycine (Gly), Leucine (Leu), tyrosine (Tyr.), lysine (Lys), L-phenylalanine (Phe), L-arginine (Arg), L-proline (Pro) and L-serine (Ser) [17]. The most abundant amino acids in wolfberry fruits are proline and serine, and the essential amino acids account for 30% of the total free amino acids [18]. In addition, wolfberry fruits are characterized by non-protein amino acids, such as gamma-aminobutyric acid, hydroxyproline and citrulline, which have specific metabolic functions [19]. Although many metabolites in numerous organs of wolfberry have been identified, very few have been linked to its physiological functions [6,20,21,22]. In addition, a large number of alkaloids, organic acids and vitamins have been identified in the fruits and roots of *Lycium barbarum* [20].

*Lycium ruthenicum* (also known as black wolfberry) is a close relative of *Lycium barbarum*, a wild perennial spiny shrub native to northwestern China [23]. Despite the close phylogenetic relationship between *Lycium barbarum* and *Lycium ruthenicum*, the fruits of these two species show different phenotypic characteristics, including shape, size, color, taste and nutritional value at different developmental stages [24,25,26]. Moreover, *Lycium ruthenicum* shows extreme salt tolerance because it reduces soil salinity [27]. However, the difference in metabolites between *Lycium barbarum* and *Lycium ruthenicum* needs to be explored, and the molecular mechanism leading to the phenotypic difference between *Lycium barbarum* and *Lycium ruthenicum* is still unclear. With the development of sequencing techniques, a high-quality genome of *Lycium barbarum* has been reported [28]. The completeness of *Lycium barbarum* and *Lycium ruthenicum* genome annotation reached 93.16% and 89.38%, containing 33,581 and 32,711 protein-coding genes, respectively [28]. A report on the *Lycium barbarum* genome will allow for the possibility of identifying the functional genes of *Lycium barbarum*.

In this study, fruits of *Lycium barbarum* and *Lycium ruthenicum* were collected at five developmental stages, from young fruits (about 10 days after flowering) to mature (ripe) fruits (34–45 days after flowering), and their metabolome and transcriptome were analyzed. Through the analysis of metabolites of *Lycium ruthenicum* and *Lycium barbarum* fruits at different developmental stages, it was found that amino acids, ABA, vitamins, sucrose and flavonoids had specific accumulation patterns at the development stage of wolfberry fruits, and these metabolites also showed differences between *Lycium ruthenicum* and *Lycium barbarum* at the same fruit development stage. The key genes involved in the metabolism pathway of flavonoids and thiamine in wolfberry fruits were identified by metabolome–transcriptome analysis, and the molecular basis of the difference in flavonoids and thiamine contents in two kinds of wolfberry was revealed.

## 2. Materials and Methods

### 2.1. Fruit Sampling

We divided the fruit maturity of *Lycium barbarum* and *Lycium ruthenicum* into five stages and collected samples at the following times after flowering: S1, young fruit (9–12 days); S2, green fruit (14–19 days); S3, coloring fruit (20–26 days); S4, immature fruit (30–37 days); and S5, mature fruit (34–45 days). After taking the samples, they were frozen in liquid nitrogen and brought back to the laboratory for lyophilization.

### 2.2. Extraction of Metabolites

The freeze-dried samples were ground for 1 min at 30 Hz using a grinder (MM 400, Retsch). Then, 100 mg of powder was weighed, and pure methanol containing 0.1 mg/L lidocaine was added for the extraction of fat-soluble metabolites (or 70% methanol was used for the extraction of water-soluble metabolites), vorticed once every 10 min three times, then stored in a refrigerator at 4 °C overnight. The next day, the sample was centrifuged (4 °C, 10,000 rpm, 10 min), and the supernatant was absorbed. The water-soluble and fat-soluble metabolites were mixed 1:1, filtered through a microporous filter membrane (SCAA-104, 13 mm, 0.22 μm, Shanghai Anpu Experimental Technology Co., Ltd., Shanghai, China, http://www.anpel.com.cn/ 15 November 2020) and stored in injection bottles for UPLC-MS analysis [29].

### 2.3. Detection of Metabolites

Metabolome data derived from the UPLC-Q-Trap 6500+ MS extensively targeted detection of metabolites. HPLC conditions were as follows. A Shimadzu C18 column (100 mm × 2.1 mm, 1.7 μm) was used as the analytical column. The column temperature was set at 40 °C. The liquid-phase flow rate was set at 0.35 mL/min. Mobile phase A was deionized water (containing 0.04% acetic acid), and mobile phase B was acetonitrile (containing 0.04% acetic acid). Elution gradient: 0 min, 5% phase B, 10 min, 95% phase B, 11 min, 95% phase B, 11.1 min, 5% phase B, 14 min, 5% phase B. The injection volume of the HPLC injector was set at 2 μL.

Electron spray ionization (ESI) was selected in mass spectrometry, and metabolites were detected in multiplex reaction monitoring mode, including positive ion mode and negative ion mode. Source/gas parameters: positive ion mode: curtain gas (CUR) set at 20 arb; ion spray (IS) voltage was set to 4500 eV; temperature (TEM) was set to 500 °C; ion source gas 1 (GS1) was set to 50 arb; and GS2 was set to 60 arb. Negative ion mode: curtain gas (CUR) WAS set to 35 arb; ion spray (IS) voltage was set to −4500 eV; temperature (TEM) was set to 500 °C, ion source gas 1 (GS1) was set to 50 arb; and GS2 was set to 60 arb. The MRM selection detection window was set to 60 s, and the target cycle time was fixed at 0.8 s. The raw data were integrated with Multi Quant 3.0.3 to accurately obtain the relative content of each substance.

### 2.4. Source and Analysis of Transcriptome Data

Transcriptome sequencing data were obtained from the NCBI Gene Expression Omnibus database (GEO) under the accession numbers GPL25820 (LB) and GPL25821 (LR) [30]. FastQC software was used to control the quality of the fastq sequence files. Fastp software was used to remove the adapter in the sequence and filter the low-quality sequence. The filtered fasta sequence was aligned to the reference genome using hisat2 software. FeaturesCounts software was used to accurately count reads. The expression level of each gene in each sample was measured according to the calculation formulas of FPKM and TPM. Through the Student’s t-test and multiple screening differences genes, the screening threshold was set to a *p*-value < 0.05 and |log_2_fold change| > 1.

### 2.5. Principal Component Analysis Based on Metabolites and Genes

The Factoextra R package was used for principal component analysis of metabolites and genes. The results showed that ggplot2 R package was used.

### 2.6. GO and KEGG Enrichment for Differentially Expressed Genes

Functional enrichment analyses, including gene ontology (GO) and the Kyoto Encyclopedia of Genes and Genomes (KEGG), were performed to identify differentially expressed genes (DEGs) that were significantly enriched in GO terms and metabolic pathways at Bonferroni-corrected *p*-values ≤ 0.05 compared with the whole-transcriptome background. GO functional enrichment and KEGG pathway analysis were performed using the clusterProfiler R package [31].

### 2.7. Coexpression Network Analysis for the Construction of Modules

Using R package weighted gene coexpression network analysis (WGCNA), gene expression in the network analysis, |*r*| ≥ 0.5 and *p*-values < 0.001 as the filter threshold, we constructed the high expression of gene modules [32,33]. WGCNA network construction and module detection were conducted using an unsigned topological overlap matrix (TOM), a power β of 19, a minimum module size of 30 and a branch merge cut height of 0.25. The correlation between metabolites and gene modules was calculated using the ggcor R package.

### 2.8. Heat Maps of Metabolite Content

Heat maps of metabolite content were generated using the heatmap package in R, homogenizing the metabolite content according to the direction of the rows [34].

## 3. Results

### 3.1. Analysis of Metabolic Profiling between Lycium barbarum and Lycium ruthenicum Fruits

**Figure 1:**
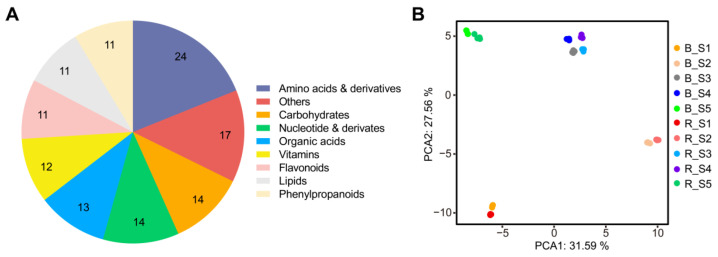
Metabolomic analysis of two wolfberry fruits. (**A**) Classes of the metabolites with annotated structures. Metabolites are divided into nine categories. (**B**) Principal component analysis of the metabolome of wolfberry fruit. B represents *Lycium ruthenicum*, R represents *Lycium barbarum* and S1–S5 represent the five stages of wolfberry fruit development.

To determine the metabolite differences between *Lycium barbarum* and *Lycium ruthenicum*, we constructed a metabolome database of wolfberry fruits using a broadly targeted metabolomics approach. The structures of 127 metabolites were identified by standards and databases, including 24 amino acids and derivatives, 14 carbohydrates, 14 nucleotides and derivates, 13 organic acids, 12 vitamins, 11 flavonoids, 11 lipids, 11 phenylpropanoids and 17 others (Figure 1A, Table S1). Principal component analysis of the metabolites revealed that the metabolomes of *Lycium barbarum* and *Lycium ruthenicum* at different fruit development stages were divided into different groups, in which the interpretation rate of the first principal component (PCA1) was 23.01%, whereas that of the second principal component (PCA2) was 19.51% (Figure 1B). These results indicate significant differences in metabolite accumulation between *Lycium barbarum* and *Lycium ruthenicum* during different fruit development periods.

### 3.2. Analysis of Metabolite Changes in Different Fruit Development Stages of Lycium barbarum and Lycium ruthenicum

**Figure 2:**
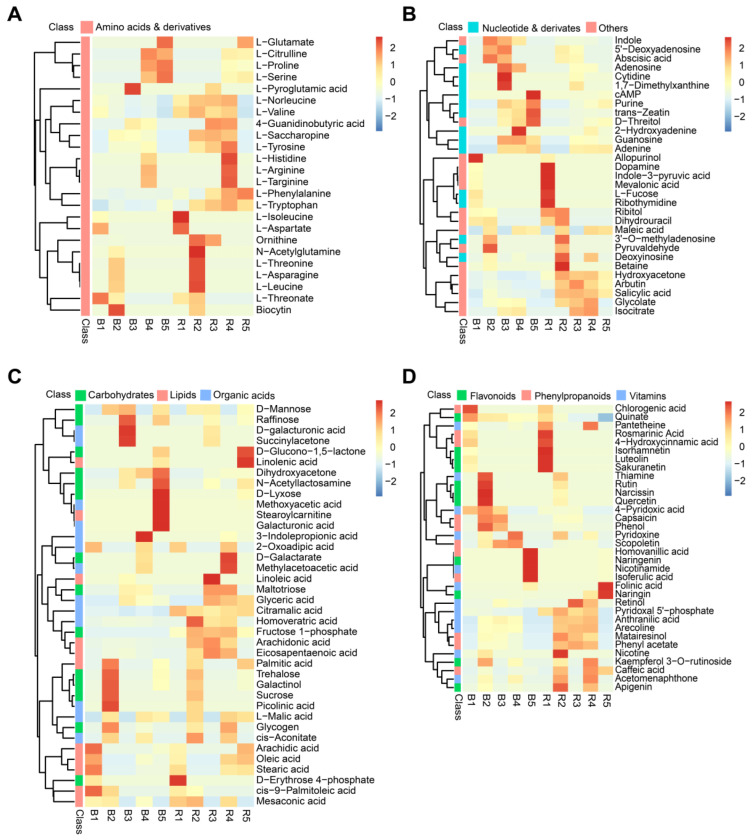
Analysis of metabolite content in fruits of *Lycium barbarum* and *Lycium ruthenicum* at different developmental stages. B1–B5 represent the five developmental stages of *Lycium ruthenicum*, and R1–R5 represent the five developmental stages of *Lycium barbarum* fruit. (**A**) Heat map of the contents of amino acids and derivatives of *Lycium barbarum* and *Lycium ruthenicum* fruits at different stages of development. (**B**) Heat map of the contents of nucleotides and derivatives and other components of *Lycium barbarum* and *Lycium ruthenicum* fruits at different stages of development. (**C**) Heat map of the contents of carbohydrates, lipids and organic acid contents of *Lycium barbarum* and *Lycium ruthenicum* fruits at different stages of development. (**D**) Heat map of the contents of flavonoids, phenylpropanoids and vitamin contents of *Lycium barbarum* and *Lycium ruthenicum* fruits at different developmental stages.

In order to investigate the changes in metabolites during the different fruit development periods of *Lycium barbarum* and *Lycium ruthenicum*, we show the metabolite contents during the different fruit development periods using a heat map (Figure 2). The results show that some amino acids have a similar accumulation pattern at the five fruit development stages in both wolfberry fruits. For example, L-isoleucine and L-aspartate accumulated during the first stage of fruit development, whereas L-threonine, L-asparagine and L-leucine accumulated during the second stage of fruit development (Figure 2A). L-proline, L-serine and L-glutamate accumulated at the fourth and fifth stages of fruit development (Figure 2A). However, the contents of these amino acids in the two kinds of wolfberry were significantly different (Figure 2A). The contents of L-proline, L-serine and L-glutamic acid were higher in *Lycium ruthenicum* than in *Lycium barbarum* during the fourth and fifth stages of fruit development, while the contents of L-isoleucine, L-aspartic acid, L-aspartic acid and L-leucine were higher in *Lycium barbarum* than in *Lycium ruthenicum* during the first and second stages of fruit development.

L-valine, L-phenylalanine and L-tryptophan showed different accumulation patterns during the development of the two kinds of wolfberry fruit. The content of L-valine at the first, second, third and fourth stages of fruit development was higher in *Lycium barbarum* than in *Lycium ruthenicum* (Figure 2A). The content of L-phenylalanine was higher in *Lycium barbarum* than in *Lycium ruthenicum* during the third, fourth and fifth stages of fruit development (Figure 2A). The content of L-tryptophan was higher in *Lycium barbarum* than in *Lycium ruthenicum* during the second, third, fourth and fifth stages of fruit development (Figure 2A). ABA is an important plant hormone that regulates plant growth, development and stress response [35]. We found that ABA accumulates specifically at the second and third stages of fruit development of *Lycium barbarum* and *Lycium ruthenicum*, and its content is higher in *Lycium ruthenicum* (Figure 2B). Moreover, we found that fructose 1-phosphate content was higher at the fifth stage of *Lycium barbarum* fruit development, and sucrose content was higher at the second stage of *Lycium ruthenicum* fruit development (Figure 2C).

Flavonoids play a crucial role in mediating plant responses to biotic and abiotic stresses [36,37]. Quercetin content was higher at the fifth stage of *Lycium ruthenicum* fruit development, and naringenin content was higher at the second stage of *Lycium ruthenicum* fruit development (Figure 2D). Kaempferol 3-*o*-rutinoside content was higher at the second stage of *Lycium ruthenicum* fruit development and at the fourth stage of fruit development of *Lycium barbarum* (Figure 2D).

Plants are an important source of vitamins for humans and are essential for their growth and development [38]. Thiamine was specifically accumulated at the first stage of fruit development of *Lycium ruthenicum*, and its content was higher in *Lycium ruthenicum* than in *Lycium barbarum* (Figure 2D). Pyridoxine and pyridoxal 5’-phosphate are both members of the vitamin B6 group [39]. Pyridoxine content was higher at the second stage of fruit development of *Lycium barbarum* and at the fourth stage of fruit development of *Lycium ruthenicum* (Figure 2D). Pyridoxal 5’-phosphate accumulates specifically from the first to fourth stages of fruit development in *Lycium barbarum*, (Figure 2D). Interestingly, Folinic acid is highly accumulated during the final stages of fruit development in *Lycium barbarum*. This means that more folic acid can be consumed by consuming mature *Lycium barbarum* fruits. In conclusion, amino acids, ABA, sucrose and vitamin metabolites not only have different accumulation patterns at the development stages of the two kinds of wolfberry fruits but also have different contents in the two kinds of wolfberry fruits at the same development stage.

### 3.3. Transcriptome Analysis of Lycium barbarum and Lycium ruthenicum

**Figure 3:**
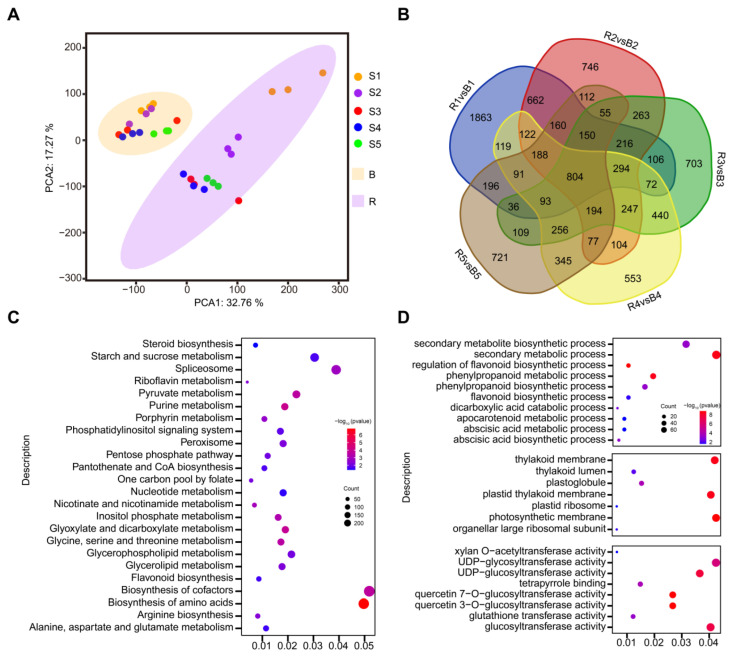
Transcriptome analysis of two kinds of wolfberry fruits. (**A**) Transcriptome principal component analysis of wolfberry fruit. B represents *Lycium ruthenicum*, R represents *Lycium barbarum* and S1–S5 represent the five stages of fruit development of wolfberry. (**B**) Gene analysis of different developmental stages of two wolfberry fruits. R1vsB1 is the differential gene between *Lycium barbarum* and *Lycium ruthenicum* at the first stage of fruit development. R2vsB2 is the differential gene between *Lycium barbarum* and *Lycium ruthenicum* at the second stage of fruit development. R3vsB3 is the differential gene between *Lycium barbarum* and *Lycium ruthenicum* at the third stage of fruit development. R4vsB4 is the differential gene between *Lycium barbarum* and *Lycium ruthenicum* at the fourth stage of fruit development. R5vsB5 is the differential gene between *Lycium barbarum* and *Lycium ruthenicum* at the fifth stage of fruit development. (**C**) KEGG enrichment analysis of different genes in different developmental stages of two wolfberry fruits. The Y axis represents KEGG pathways, and the X axis indicates the gene ratio. (**D**) GO enrichment analysis of different genes in different developmental stages of two wolfberry fruits. The Y axis represents GO pathways, and the X axis indicates the gene ratio.

In order to explore the genetic basis of metabolite content differences between *Lycium barbarum* and *Lycium ruthenicum*, we analyzed the transcriptomes of *Lycium barbarum* and *Lycium ruthenicum* fruit at different developmental stages (Table S2). The results of PCA indicate that the transcriptomes of *Lycium barbarum* and *Lycium ruthenicum* were divided into two sets at different fruit development stages, in which the interpretation rate of the first principal component (PCA1) was 32.76%, and that of the second principal component (PCA2) was 17.27% (Figure 3A). The results suggest that the gene expression of *Lycium barbarum* and *Lycium ruthenicum* was different at the same stage of fruit development. To further verify the above results, we analyzed the genes of *Lycium barbarum* and *Lycium ruthenicum* at different fruit development stages. There were 5171 differentially identified genes at the first stage, 4393 differentially identified metabolites at the second stage, 4037 differentially identified genes at the third stage, 3998 differentially identified genes at the fourth stage and 3586 differentially identified genes at the fifth stage of the *Lycium barbarum* and *Lycium ruthenicum* (Figure 3B). We also performed KEGG and GO enrichment analysis for all differential genes. The results show that the flavonoid synthesis pathway was enriched (Figure 3C). The results of GO analysis also indicate an enriched flavonoid synthesis pathway (Figure 3C). These results suggest that there are differences in the expression of flavonoid synthesis pathway genes between *Lycium barbarum* and *Lycium ruthenicum* at different stages of fruit development.

### 3.4. Correlation Networks Based on Genes and Metabolites during Fruit Development of Wolfberry

**Figure 4:**
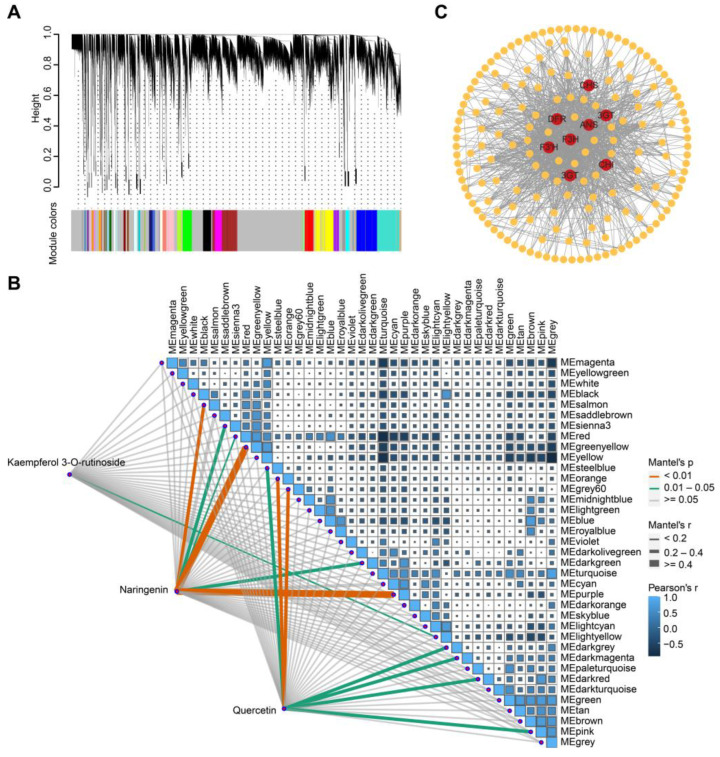
Correlation network analysis of metabolites and genes in wolfberry during fruit development. (**A**) Construct coexpression modules of genes. The same colors represent the same modules. (**B**) Correlation analysis between metabolites and gene modules. (**C**) Network analysis of genes in modules. Through network analysis, the core genes in the modules with significant correlation with metabolites were identified. The identified genes are as follows: *CHS*, *chalcone synthase*; *CHI*, *chalcone isomerase*; *F3H*, *flavanone 3-hydroxylase*; *F3′H*, *flavonoid 3′-hydroxylase*; *DFR*, *dihydroflavonol reductase*; *ANS*, *anthocyanidin synthase*; *3GT*, *flavonoid 3-O-glucosyltransferase*.

To decipher the molecular basis of metabolite differences during the development of *Lycium barbarum* and *Lycium ruthenicum* fruit, a WGCNA analysis was conducted based on the transcriptome data of five developmental stages of two kinds of wolfberry fruits. The results indicate that the transcriptome data were divided into 37 gene modules (Figure 4A). The genes in each module have similar expression patterns, and these genes may be involved in the same pathway. Correlation analysis of 37 gene modules with naringenin, quercetin and kaempferol 3-*o*-rutinoside showed that naringenin was significantly correlated with MEgreenyellow and MEpurple (*r* ≥ 0.4, *p* < 0.01) (Figure 4B). These results indicate that there may be genes involved in flavonoid synthesis in these gene modules. A network analysis of genes in MEgreenyellow and MEpurple gene modules was conducted to identify the core genes in the modules, and a homologous sequence alignment of the identified core genes was conducted.. The results show that some key genes of the flavonoid synthesis pathway were identified from the core genes of the gene module, such as *chalcone synthase* (*CHS*), *chalcone isomerase* (*CHI*), *flavanone 3-hydroxylase* (*F3H*) and *flavonoid 3’-hydroxylase* (*F3’H*) (Figure 4C). By combining metabolome and transcriptome analysis, we identified possible genes involved in the flavonoid synthesis pathway in wolfberry fruits.

### 3.5. Genetic Mechanism of Flavonoid Metabolite Content Difference in Fruits of Lycium barbarum and Lycium ruthenicum

**Figure 5:**
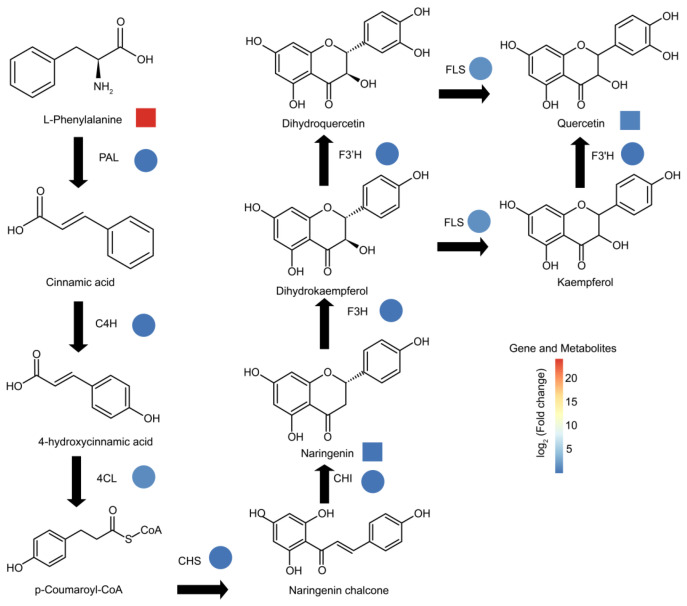
Pathways of flavonoid synthesis in fruits of *Lycium barbarum* and *Lycium ruthenicum*. The heat map represents the expression multiples and metabolite content multiples of corresponding structural genes in *Lycium barbarum* and *Lycium ruthenicum*. The heat map shows the expression level of structural genes and metabolite contents from low to high, ranging from blue to red. Genes involved in flavonoid synthesis are shown as follows: *PAL*, *phenylalanine ammonia lyase*; *C4H*, *cinnamate 4-hydroxylase*; *4CL*, *4-coumarate CoA ligase*; *CHS*, *chalcone synthase*; *CHI*, *chalcone isomerase*; *F3H*, *flavanone 3-hydroxylase*; *F3′H*, *flavonoid 3′-hydroxylase*.

To investigate the genetic basis of the difference between flavonoid metabolites in fruits of *Lycium barbarum* and *Lycium ruthenicum*, we mapped the pathway of flavonoid synthesis and analyzed gene expression levels and metabolite contents related to flavonoid synthesis in fruits of *Lycium barbarum* and *Lycium ruthenicum*. We found that the expression levels of key genes involved in flavonoid synthesis, such as *phenylalanine ammonia lyase* (*PAL*), *cinnamate 4-hydroxylase* (*C4H*), *4-coumarate CoA ligase* (*4CL*), *chalcone synthase* (*CHS*), *chalcone isomerase* (*CHI*), *flavanone 3-hydroxylase* (*F3H*), *flavonoid 3′-hydroxylase* (*F3′H*) and *flavonol synthase* (*FLS*), were higher in *Lycium ruthenicum* fruits than in *Lycium barbarum* fruits (Figure 5, Table S3). We also detected significant differences in some metabolites of the flavonoid synthesis pathway between the two kinds of wolfberry fruits. The content of phenylalanine, the starting metabolite of the flavonoid synthesis pathway, is higher in fruits of *Lycium barbarum* than in fruits of *Lycium ruthenicum* (Figure 5). The contents of naringenin and quercetin, two flavonoid metabolites, were higher in fruits of *Lycium ruthenicum* than in *Lycium barbarum* (Figure 5). These results suggest that the flavonoid difference between *Lycium ruthenicum* and *Lycium barbarum* may be caused by the higher expression levels of *PAL*, *C4H*, *4CL*, *CHS*, *CHI*, *F3H*, *F3’H* and *FLS* genes in *Lycium ruthenicum* fruits.

## 4. Discussion

In this study, we analyzed the intraspecific and interspecific metabolome changes of two species of wolfberry at different fruit development stages and found that significant changes occurred in both intraspecific and interspecific metabolomes during fruit development. In particular, the contents of nutritional metabolites and flavor metabolites, such as amino acids and derivatives, vitamins, flavonoids and sugars, were significantly different during the development of the two kinds of wolfberry fruits. These results indicate that the two kinds of wolfberry were different in flavor and nutritional value. It was found that *Lycium barbarum* contained higher levels of phenylalanine, and *Lycium ruthenicum* contained higher levels of naringenin and quercetin. The key genes in the pathway of flavonoid synthesis were identified by the combined analysis of the metabolome and transcriptome of the two fruits at different developmental stages, such as *PAL*, *C4H*, *4CL*, *CHS*, *CHI*, *F3H*, *F3’H* and *FLS*.

**Figure 6:**
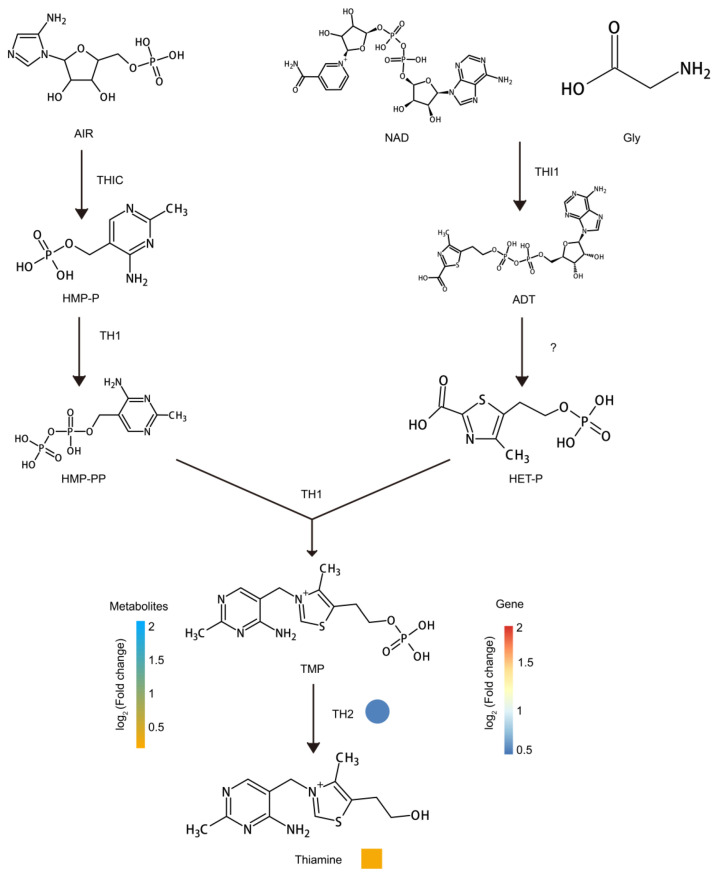
Pathway of thiamine synthesis in fruits of *Lycium barbarum* and *Lycium ruthenicum*. The heat map represents the expression multiples and metabolite content multiples of corresponding structural genes in *Lycium barbarum* and *Lycium ruthenicum*. The heat map shows the expression level of structural genes from blue to red and from low to high. The heat map shows the metabolite content from yellow to blue and from low to high. Genes involved in flavonoid synthesis are shown as follows: *ADT*: *adenylated thiazole*; *AIR*: *5-aminoimidazole ribonucleotide*; *HET-P*: *hydroxyethyl thiazole phosphate*; *HMP-P*: *4-amino-2-methyl-5-hydroxyl methyl pyrimidine monophosphate*; *HMP-PP*: *4-amino-2-methyl-5-hydroxymethyl pyrimidine diphosphate*; *NAD*: *nicotinamide adenine dinucleotide*; *TH1*: *thiamine phosphate synthase*; *TH2*: *thiamine monophosphate phosphatase*; *THI1*: *thiazole synthase*; *THIC*: *pyrimidine synthase*; *TMP*: *thiamine monophosphate*.

Sugar is the main source of fruit sweetness and an important component of fruit flavor, and fruit with a moderately higher sugar content is favored by consumers [40,41]. The sucrose content in *Lycium ruthenicum* is higher during the second stage of wolfberry development (Figure 2C). As far as we know, *Lycium barbarum* is sweeter than *Lycium ruthenicum* [42]. Therefore, the sweetness of wolfberry is not determined by the sucrose content alone but by other metabolites related to sweetness. Fructose is a type of sugar with a sweet taste [43]. The content of fructose 1-phosphate is higher in *Lycium barbarum* (Figure 2C). Due to the lack of human nutrition, nutrition obtained from fruits and vegetables has been a worldwide concern. Studies have shown that there are differences in nutrient composition at different stages of plant development; for example, carotenoids, such as violet xanthin, neoxanthin and vitamin K1, are found at higher levels in seedlings of *Brassica rapa* subsp. *chinensis* var. *parachinensis* [44,45]. Similarly, a variety of nutrients, such as amino acids and vitamins, have been detected in the fruits of wolfberries [14,18,46,47,48]. In amino acid analysis, we found that some amino acids had similar accumulation patterns at the development stage of two kinds of wolfberry fruits, but their contents were different in the two kinds of wolfberry fruits at the same development stage, such as L-glutamate, L-proline, L-serine, L-isoleucine, L-aspartate, L-threonine, L-asparagine and L-leucine (Figure 2A). Other amino acids showed different accumulation patterns in two kinds of *Lycium barbarum* fruits at the same developmental stage, and their contents were also different in two kinds of wolfberry fruits, such as L-valine, L-phenylalanine and L-tryptophan (Figure 2A). Specific accumulation of amino acids in different developmental stages of wolfberry fruits suggests the complexity of amino acid biosynthesis during the development of wolfberry. The accumulation of vitamins also showed significant differences at different stages of fruit development in wolfberry. For example, it has been reported that the fruits of wolfberry contain rich thiamine [49]. We found that thiamine content was highest at the second stage of fruit development of the two kinds of wolfberry, and the content of *Lycium ruthenicum* was higher than that of *Lycium barbarum* (Figure 6). We also analyzed the transcriptome data and found that the expression level of *thiamine monophosphate phosphatase* (*TH2*) in *Lycium ruthenicum* was higher than that in *Lycium barbarum* at the second stage of fruit development (Figure 6, Table S4). We found that *TH2* expression was highest during the second stage of fruit development in both wolfberry types, which explains the highest accumulation of thiamin acquired during the second stage of fruit development.

ABA participation in plant salt stress response can activate the expression of salt-stress-response genes and improve plant salt tolerance [50,51,52]. Many species of the genus *Lycium* have a high salt tolerance [53,54]. In this study, we found that ABA specifically accumulated at the second and third stages of fruit development of both kinds of wolfberry (Figure 2B). The possible reason for this result is that it is mainly distributed in the high-salinity soil in the northwest of China [27]. The content of ABA was higher in *Lycium ruthenicum* than in *Lycium barbarum* (Figure 2B). *Aldehyde oxidase (AAO)* is one of the genes involved in ABA biosynthesis [35]. Its expression in *Lycium ruthenicum* is higher than that in *Lycium barbarum* (Table S5). These results are consistent with previous reports stating that *Lycium ruthenicum* showed higher salt tolerance than *Lycium barbarum* [55]. We also noted that trans-zeatin content was highest at the fifth stage of *Lycium ruthenicum* fruit development, and 3-indolepropionic acid (IPA) content was highest at the first stage of *Lycium barbarum* fruit development (Figure 3A). However, the link between these hormones and salt tolerance in *Lycium barbarum* and *Lycium ruthenicum* needs to be further investigated.

The fruits of wolfberry contain high flavonoid metabolite contents [56]. Flavonoids have the function of promoting digestion and enhancing immunity [57] and are subject to different accumulation rules in different varieties of the same fruit [58]. Flavonoids can prevent chronic diseases, and flavonoid-rich plants can increase tolerance to biological or abiotic stresses [59,60]. We found that the pathway of flavonoid synthesis was significantly different in the two kinds of wolfberry fruits. There are obvious differences in color between *Lycium barbarum* and *Lycium ruthenicum*, and there are significant differences in anthocyanin type and content between the two kinds of wolfberry [61]. In many plant fruits, genes involved in anthocyanin biosynthesis belong to the flavonoid synthesis pathway [62]. The difference in gene expression and flavonoid content in the flavonoid synthesis pathway of two kinds of wolfberry affected the synthesis of anthocyanin in the downstream, which resulted in the difference in color between the two kinds of wolfberry.

In this study, metabolomics methods were used to analyze the metabolite differences between *Lycium barbarum* and *Lycium ruthenicum* at different fruit development stages, with particular emphasis on the different metabolites related to flavor and nutrition (sugars, amino acids, vitamins and flavonoids) in wolfberry fruits. Through the combined analysis of the metabolome and transcriptome, the possible genetic basis of different metabolites was analyzed, providing new insights into the metabolic mechanism of the flavor and nutritional quality of wolfberry.

## Data Availability

The data presented in this study are available on request from the corresponding authors. The data are not publicly available due to privacy.

## Supplementary Materials

The following supporting information can be downloaded at: https://www.mdpi.com/article/10.3390/metabo13060680/s1, Table S1. Data matrix of metabolite signatures in wolfberry fruits. Table S2. Gene expression data matrix of wolfberry fruits. Table S3. The expression levels of genes related to flavonoid biosynthesis in wolfberry fruits. Table S4. Expression level of *TH2* in wolfberry fruits. Table S5. Expression level of *AAO3* in wolfberry fruits.
